# Does Vitamin D matter? The role of calcium homeostasis imbalance in clinical severity of psychiatric patients

**DOI:** 10.1192/j.eurpsy.2022.713

**Published:** 2022-09-01

**Authors:** G. Altucci, L. Giannelli, C. Palummo, A. Di Cerbo, S. Agnese, S. Cipolla, M. Fabrazzo, F. Perris, F. Catapano, M. Luciano, G. Sampogna, A. Fiorillo

**Affiliations:** University of Campania “Luigi Vanvitelli”, Department Of Psychiatry, Naples, Italy

**Keywords:** Vitamin D, Clinical severity, Calcium homeostasis, Psychiatric patients

## Abstract

**Introduction:**

Vitamin D modulates the biosynthesis of neurotransmitters and neurotrophic factors and it is involved in the modulation of inflammatory responses, with a potential impact on clinical status of patients with severe mental disorders. Moreover, available evidences report that decreased blood levels of Vitamin D are associated to a worse course of psychotic and affective disorders.

**Objectives:**

We assessed calcium homeostasis imbalance in a sample of inpatients and outpatients, referring to the Department of Psychiatry of University of Campania “Luigi Vanvitelli” in order to explore levels of Calcium, PTH and Vitamin D and their influence in clinical severity among this different subgroups.

**Methods:**

All patients were administered The Brief Psychiatric Rating Scale (BPRS) to assess different domains of psychopathology. Vitamin D, Calcium and PTH levels were assessed in all patients. An-ad hoc schedule was administered for socio-demographic and clinical characteristics.

**Results:**

The total sample consisted of 152 patients (75 males and 77 females with 47.3 ± 14.4 age at admission, 74 inpatients and 78 outpatients). Patients with lower level of Vitamin D are more likely to present higher number of relapses (p<0.05) and to be inpatients (<0.01). Finally, serum levels of Vitamin D were negatively correlated with all the BPRS subscales (p < 0.01).

**Conclusions:**

Lower levels of Vitamin D correlate with a worse clinical outcome of patients with different psychiatric diagnosis. Our results highlight the importance to routinely assess PTH, Vit D and calcium levels, especially in inpatients. Moreover, Vitamin D may represent a valid add-on treatment for these patients.

**Disclosure:**

No significant relationships.

